# Saturated free fatty acids induce placental trophoblast lipoapoptosis

**DOI:** 10.1371/journal.pone.0249907

**Published:** 2021-04-22

**Authors:** Sathish Kumar Natarajan, Taylor Bruett, Philma Glora Muthuraj, Prakash K. Sahoo, Jillian Power, Justin L. Mott, Corrine Hanson, Ann Anderson-Berry

**Affiliations:** 1 Department of Nutrition and Health Sciences, University of Nebraska-Lincoln, Lincoln, NE, United States of America; 2 Department of Biochemistry and Molecular Biology, University of Nebraska Medical Center, Omaha, NE, United States of America; 3 College of Allied Health Professions Medical Nutrition Education, University of Nebraska Medical Center, Omaha, NE, United States of America; 4 Department of Pediatrics, University of Nebraska Medical Center, Omaha, NE, United States of America; University of Illinois, UNITED STATES

## Abstract

**Introduction:**

Obesity during pregnancy increases the risk for maternal complications like gestational diabetes, preeclampsia, and maternal inflammation. Maternal obesity also increases the risk of childhood obesity, intrauterine growth restriction (IUGR) and diabetes to the offspring. Increased circulating free fatty acids (FFAs) in obesity due to adipose tissue lipolysis induces lipoapoptosis to hepatocytes, cholangiocytes, and pancreatic-β-cells. During the third trimester of human pregnancy, there is an increase in maternal lipolysis and release of FFAs into the circulation. It is currently unknown if increased FFAs during gestation as a result of maternal obesity cause placental cell lipoapoptosis. Increased exposure of FFAs during maternal obesity has been shown to result in placental lipotoxicity. The objective of the present study is to determine saturated FFA-induced trophoblast lipoapoptosis and also to test the protective role of monounsaturated fatty acids against FFA-induced trophoblast lipoapoptosis using *in vitro* cell culture model. Here, we hypothesize that saturated FFAs induce placental trophoblast lipoapoptosis, which was prevented by monounsaturated fatty acids.

**Methods:**

Biochemical and structural markers of apoptosis by characteristic nuclear morphological changes with DAPI staining, and caspase 3/7 activity was assessed. Cleaved PARP and cleaved caspase 3 were examined by western blot analysis.

**Results:**

Treatment of trophoblast cell lines, JEG-3 and JAR cells with palmitate (PA) or stearate (SA) induces trophoblast lipoapoptosis as evidenced by a significant increase in apoptotic nuclear morphological changes and caspase 3/7 activity. We observed that saturated FFAs caused a concentration-dependent increase in placental trophoblast lipoapoptosis. We also observed that monounsaturated fatty acids like palmitoleate and oleate mitigates placental trophoblast lipoapoptosis caused due to PA exposure.

**Conclusion:**

We show that saturated FFAs induce trophoblast lipoapoptosis. Co-treatment of monounsaturated fatty acids like palmitoleate and oleate protects against FFA-induced trophoblast lipoapoptosis.

## Introduction

The prevalence of obesity is an epidemic in the United States as approximately 67% of US population is either obese or overweight [1, 2]. Sixty percent of all pregnant women in the US are predicted to be either overweight or obese [3–6]. Obese women have reproductive disorders complicating pregnancy and perinatal outcomes including ovulation defects, infertility, gestational diabetes, early pregnancy loss and birth defects [7, 8]. Pre-gravid obesity or excessive gestational weight gain has been shown to increase the risk of preeclampsia, intrauterine growth restriction (IUGR), preterm birth, stillbirth, and macrosomia [9]. Maternal obesity also increases the risk for metabolic diseases to the offspring latter in their life [4, 10–12].

Obesity or metabolic syndrome increases adipose tissue lipolysis during the overnight fasting time and leads to an increased level of FFA in the circulation [13]. Maternal adipose tissue lipolysis is enhanced in the third trimester of pregnancy and increases the flux of FFA to the fetus via the placenta, which is even greater in obese mothers [14, 15]. Recent clinical study showed that obese mothers were observed to have increased accumulation of placental lipids compared non-obese mother [16], contributing to the placental lipotoxicity and cellular stress [6, 17–19]. Palmitate is the predominant saturated fatty acid in diets and are elevated in the circulation of obese women compared to lean control [20]. Further, placental trophoblast apoptosis were also reported in obese mothers [21]. It was suggested that monounsaturated fatty acids could have a protective role against placental lipotoxicity [20, 22, 23]. However, an important gap in knowledge exists on the causal role of FFA-induced placental lipotoxicity and the protective role of mono-unsaturated fatty acids. Further, a decreased palmitate catabolism and an increased lipid droplets accumulation were observed in term placental trophoblast obtained from obese mother compared to lean mother [2]. The objective of this study is to determine saturated FFA-induced trophoblast lipoapoptosis and also to test the protective role of monounsaturated fatty acids against FFA-induced trophoblast lipoapoptosis using *in vitro* cell culture model. In the present study, we *hypothesize*d that exposure of saturated FFAs to placental trophoblast induces lipoapoptosis, which is prevented by co-treatment of monounsaturated fatty acids.

## Materials and methods

### Materials

All chemicals and buffers were analytical grade and purchased from ThermoFisher Scientific, Waltham, Massachusetts, USA. Lipopolysaccharide (LPS), palmitic acid (#P5585), stearic acid (#S4751), oleic acid (#O1008) and fatty acid-free bovine serum albumin (BSA) (#A3803) were obtained from MilliporeSigma, St. Louis, Missouri, USA.

### Antibodies

Rabbit antisera against PARP (9542S), cleaved caspase 3 (#9664) were from Cell Signaling, Danvers, Massachusetts, USA. Actin antibody (#A-5441) was from MilliporeSigma, St. Louis, Missouri, USA. Peroxidase-conjugated secondary antibodies were obtained from Jackson ImmunoResearch lab, West Grove, Pennsylvania, USA.

### Cell lines and treatment

HTR-8SV/neo (HTR-8), normal human immortalized first-trimester placental trophoblast cells, and choriocarcinoma-derived third-trimester placental trophoblast cell lines (JEG-3 and JAR) were used. JEG-3 and JAR cells has similar biochemical and biological characteristics of human term-derived trophoblasts. JAR and HTR-8 cells were cultured in DMEM containing 10% fetal bovine serum, 0.01% plasmocin and JEG-3 cells were cultured in MEM containing 10% fetal bovine serum, and 0.01% plasmocin as described [24]. All the cells used in the present study were obtained from the American type culture collection (ATCC) and periodically tested for mycoplasma.

### Fatty acid preparation

Fatty acids, palmitic acid (PA), stearic acid (SA), Oleic acid (OA) and palmitoleic acid (PO) were dissolved in isopropanol with a stock solution of 80 mM. These fatty acids were incubated with growth media containing 1% BSA for 20 minutes at 37ᵒC for fatty acid-BSA conjugation and then treated for 24 hours. BSA (1%) was dissolved in growth media at room temperature and incubated at 37ᵒC for 30 minutes prior to the addition of fatty acids. We have used various concentrations of FFAs (200–800 μM) in the present study and vehicle (Veh) treatment were <1% isopropanol in 1% BSA containing medium. The concentration range of FFAs used in the present study mimics physiological to pathophysiological concentrations.

### Measurement of apoptosis

Structural and biochemical markers of apoptosis like percent apoptotic nulcei and caspase 3/7 activity, respectively were assessed. Percent apoptotic nuclei was quantified by characteristic nuclear morphological changes and visualized by treatment with the fluorescent DNA-binding dye, DAPI (4’, 6-diamidine-2-phenylindole dihydrochloride) as described [25]. Briefly, cells were stained with 5 μg/ml of DAPI for 20–30 minutes at 37°C. Apoptotic nuclei (condensed, fragmented) were counted and presented as a percent of total nuclei. At least 200 cells were counted per well and experiments were performed in triplicate. Caspase 3/7 activity was measured using rhodamine 110 bis-(N-CBZ-I-aspartyl-I-glutamyl-I-Valyl-aspartic acid amide (Z-DEVD-R110) substrate. The caspase 3 and 7 enzyme activity in the cells will cleave the DEVD peptide in the substrate and release the rhodamine 110 fluorophore which can be measured spectrofluorometrically (Biotek Synergy) with 498nm wavelength of excitation and 521nm emission. The activity is measured by net fluorescence (assay rate of fluorescence (RFU)—negative control RFU) according to the manufacturer’s instructions (APO-One, Promega, Madison, WI #G7791). The data were reported as fold-change of net fluorescence compared to vehicle treated cells, with experiments performed in quadruplicate.

### Immunoblot analysis

Cells were rinsed once with ice-cold phosphate buffered saline (PBS) and cell lysates were prepared by scraping the cells in lysis buffer containing 50 mM tris, 150 mM sodium chloride, 1 mM ethylene diamine tetra acetic acid, 1 mM dithiothreitol, 1 mM α-phenyl methyl sulfonyl fluoride, 1 mM sodium orthovanadate, 100 mM sodium fluoride, and 1% triton X-100 along with the addition of mammalian protease inhibitor cocktail (Roche). After 40 minutes incubation on ice, whole cell lysates were centrifuged at 15,000 x *g* for 10 min to remove insoluble proteins. Cell lysates containing 25–30 μg of protein were resolved by SDS-PAGE. Proteins were transferred to a nitrocellulose membrane and visualized by immunoblotting. Briefly, transferred membrane were blocked in 5% milk powder dissolved in PBST (0.1% Tween 20 in PBS) and incubated overnight in primary antibodies with a dilution of 1 in 1000 at 4°C. After three washes for 10 minutes in PBST at room temperature, respective peroxidase-conjugated secondary antibodies were incubated for 90 minutes. The band intensity was developed using Chemidoc, Bio-Rad, Hercules, California, USA.

### Statistics

Data are expressed as mean ± standard error of mean (SEM). Statistical analysis of apoptotic nuclei levels and caspase 3/7 activity for each fatty acid was performed using one-way analysis of variance (ANOVA) with fatty acid concentration as the fixed effect and a Bonferroni *post-hoc* correction. A Bonferroni adjusted *P* value < 0.05 was accepted as statistically significant. Statistical calculations were performed using SPSS Statistics for Windows, Version 26.0. Armonk, New York: IBM Corp., USA.

## Results

### Saturated FFAs induce trophoblast lipoapoptosis

**Fig 1:**
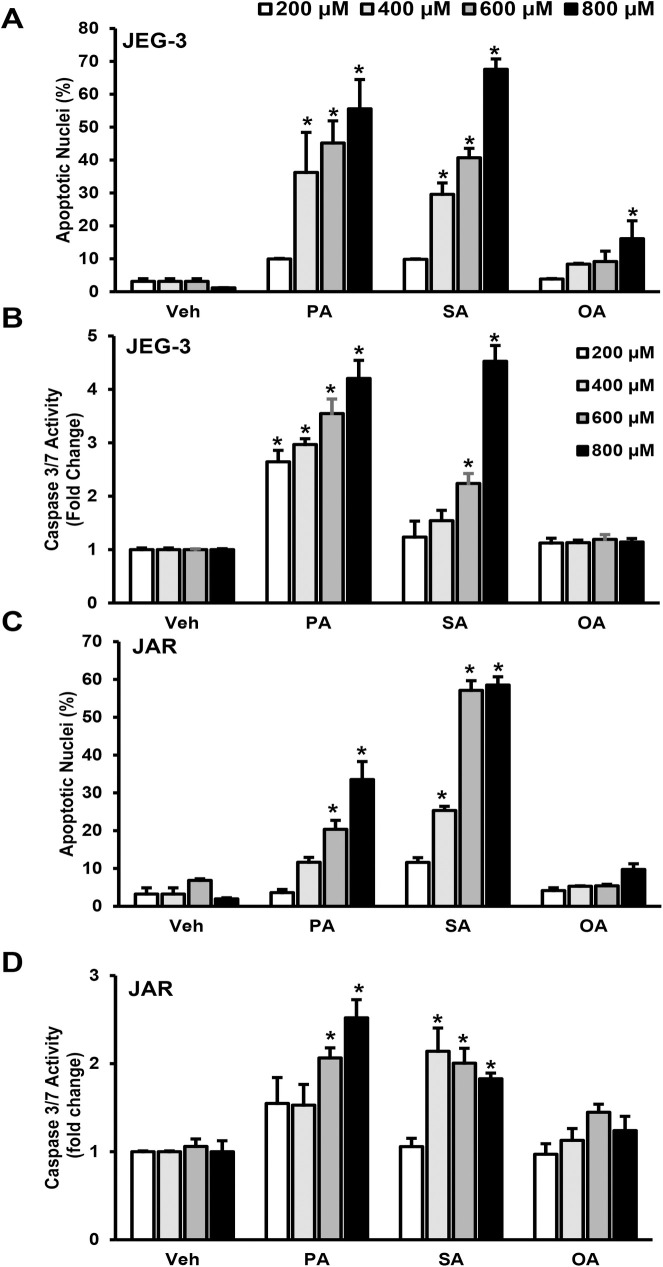
FFAs induce trophoblast lipoapoptosis. Cells were treated with palmitate (PA), stearate (SA), oleate (OA) or vehicle (Veh) for 24 h in JEG-3 (A, B) and JAR cells (C, D). Percent apoptotic nuclei (A and C) and caspase 3/7 activity (B and D) were significantly elevated in PA- and SA-treated trophoblasts but not in vehicle and OA-treated trophoblasts (A-D). These data represent the mean ± sem, n = 4 and *P<0.05 compared to vehicle.

We assessed biochemical characteristics of apoptosis like nuclear morphological changes and caspase 3/7 activity. Treatment of placental trophoblast cells, JEG-3 with saturated FFAs such as palmitate and stearate for 24 h showed trophoblast lipoapoptosis. We observed a significant increase in percent apoptotic nuclei levels and caspase 3/7 activity with increasing the concentration of palmitate or stearate (400–800 μM) treatment compared to vehicle treated cells (**Fig 1A and 1B**). In addition, treatment of 200 μM palmitate for 24 h in JEG-3 cells showed a significant increase in caspase 3/7 activity compared to vehicle treated cells, however the percent apoptotic nuclei levels were not increased with 200 μM palmitate compared to vehicle treated cells (**Fig 1A and 1B**). Treatment of oleate (200–600 μM, 24 h) to JEG-3 cells did not induce trophoblast lipoapoptosis (**Fig 1A and 1B**). However, oleate treatment did show slight but significant increase in percent apoptotic nuclear morphological changes indicating cell death only at 800 μM of oleate, highest concentration of oleate tested (**Fig 1A**). We also observed increased percent apoptotic nuclei and caspase 3/7 activity with 600–800 μM of palmitate for 24 h in JAR cells, another choriocarcinoma-derived trophoblast (**Fig 1C and 1D**). Similarly, treatment of stearate (400–800 μM) for 24 hours also showed a significant increase in percent apoptotic nuclear morphological changes and caspase 3/7 activation in JAR cells compared to vehicle treated cells (**Fig 1C and 1D**). In JAR cells, treatment of monounsaturated fatty acid, oleate (200–800μM, 24 h) did not alter the levels of percent apoptotic nuclei and caspase 3 and 7 activity compared to vehicle treated cells (**Fig 1C and 1D**). These data suggest that saturated FFAs induce trophoblast lipoapoptosis.

**Fig 2:**
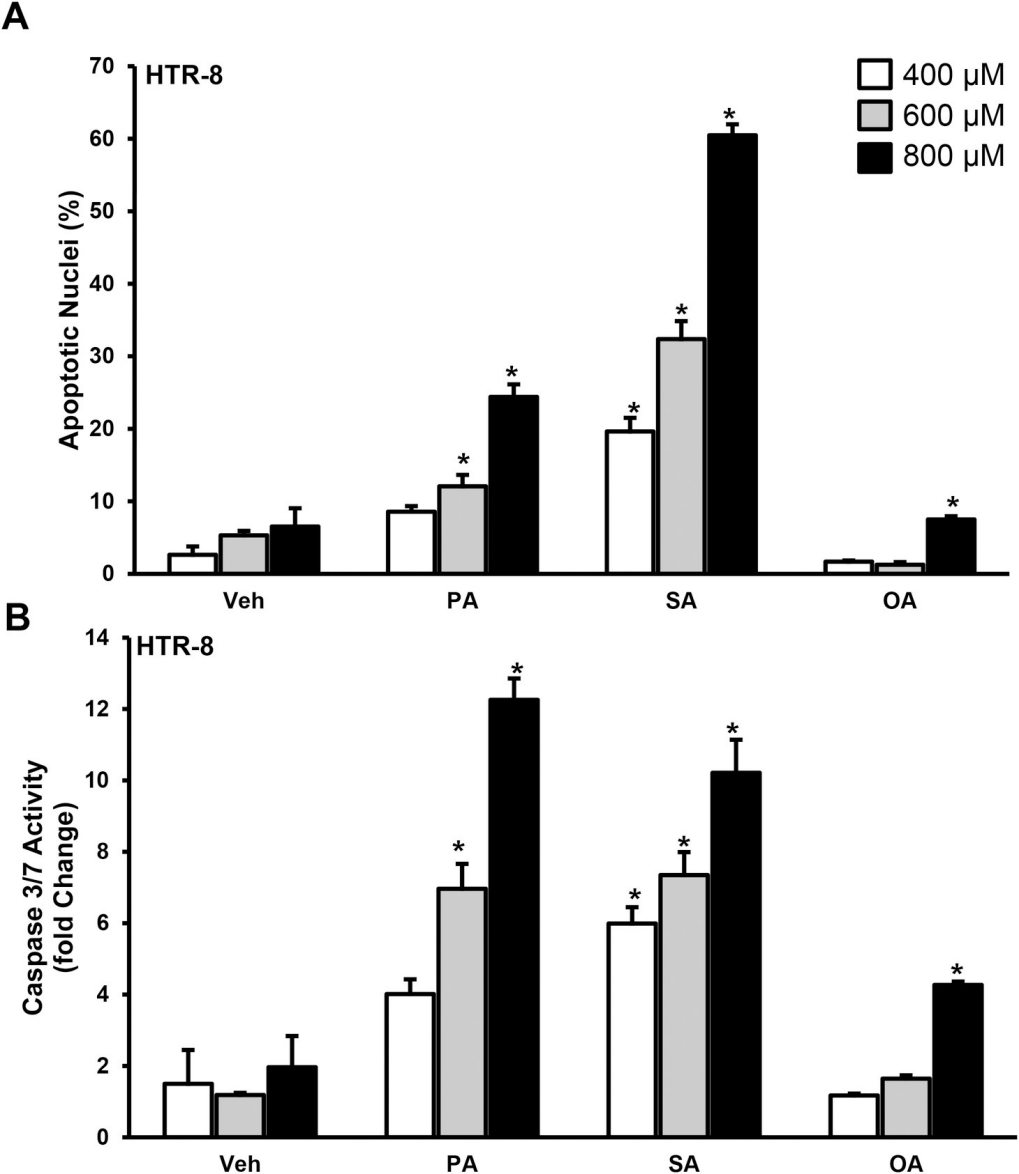
FFAs induce trophoblast lipoapoptosis in human normal immortalized first trimester-derived trophoblast cells, HTR-8SV/neo (HTR-8). HTR-8 cells were treated with palmitate (PA), stearate (SA), oleate (OA) or vehicle (Veh) for 24 hr. Percent apoptotic nuclei (A) and caspase 3/7 activity (B) were significantly elevated in PA- and SA-treated trophoblasts, but not in vehicle and OA-treated trophoblasts. These data represent the mean ± sem, n = 4 and *P<0.05 compared to vehicle.

We further used HTR-8 cells and observed that palmitate and stearate treatment for 24 h showed a concentration dependent increase in trophoblast lipoapoptosis as evidenced by an increase in percent apoptotic nuclei (**Fig 2A**) and caspase 3/7 activity (**Fig 2B**). Treatment of oleate induce lipoapoptosis only at a concentration of 800 μM. (**Fig 2A and 2B**). In summary, we observed that saturated FFAs induce a concentration-dependent increase in placental trophoblast lipoapoptosis.

### Biochemical evidence for placental trophoblast lipoapoptosis

**Fig 3:**
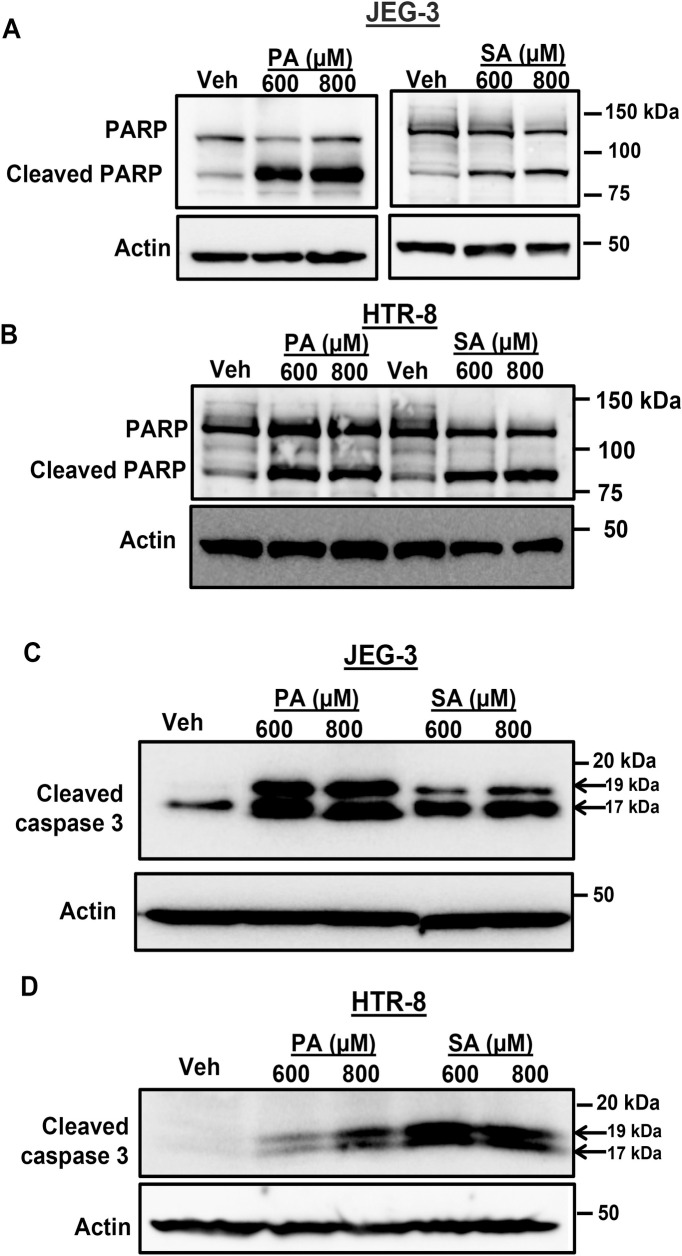
Biochemical evidence for FFA-induced trophoblast lipoapoptosis. Cells were treated with palmitate (PA), stearate (SA) (600–800 μM) or Vehicle (Veh) for 24 h. (A) JEG-3 cells treated with 600–800 μM of PA or SA showed dramatic increase in cleaved PARP levels compared to vehicle treated cells; (B) HTR-8 cells also showed increased levels of cleaved PARP with 600–800 μM of PA or SA treatment compared to vehicle (Veh) treated cells; (C) JEG-3 cells or (D) HTR-8 cells treated with 600–800 μM of PA or SA for 24 h resulted in increased levels of cleaved caspase 3 (19 kDa and 17 kDa) compared to vehicle (Veh) treated cells. Actin was used as a loading control. Arrows indicates 19 and 17 kDa bands of active forms of cleaved caspase 3.

We assessed the caspase cleavage products of Poly (ADP-ribose) polymerase (PARP) and caspase 3 itself. PARP is a 116 kDa protein with a role in DNA repair, chromatin structure formation, and differentiation [26]. Treatment of palmitate (600–800 μM) showed a dramatic increase in the levels of 85 kDa cleaved PARP in JEG-3 and HTR-8 cells after 24 hours (**Fig 3A and 3B**). We also observed a similar increase in the levels of cleaved PARP after 24 hours with stearate treatment in placental trophoblasts (**Fig 3A and 3B**). Full length caspase 3 is a 35 kDa protein and its activation occurs in two stages. First, caspase 3 is cleaved by upstream caspases to yield an active 19 kDa cleaved caspase 3. Second, a short pro-domain in the 19 kDa protein is auto-catalytically processed to generate a mature 17 kDa cleaved caspase 3 [27]. Treatment of palmitate or stearate, 600–800 μM for 24h to JEG-3 or HTR-8 cells showed a dramatic increase in the levels of both 19 kDa and 17 kDa active forms of cleaved caspase 3 compared to vehicle treated cells (**Fig 3C and 3D**). These results demonstrate further biochemical evidence for saturated FFA-induced placental trophoblast lipoapoptosis.

### LPS aggravates palmitate-induced trophoblast lipoapoptosis

**Fig 4:**
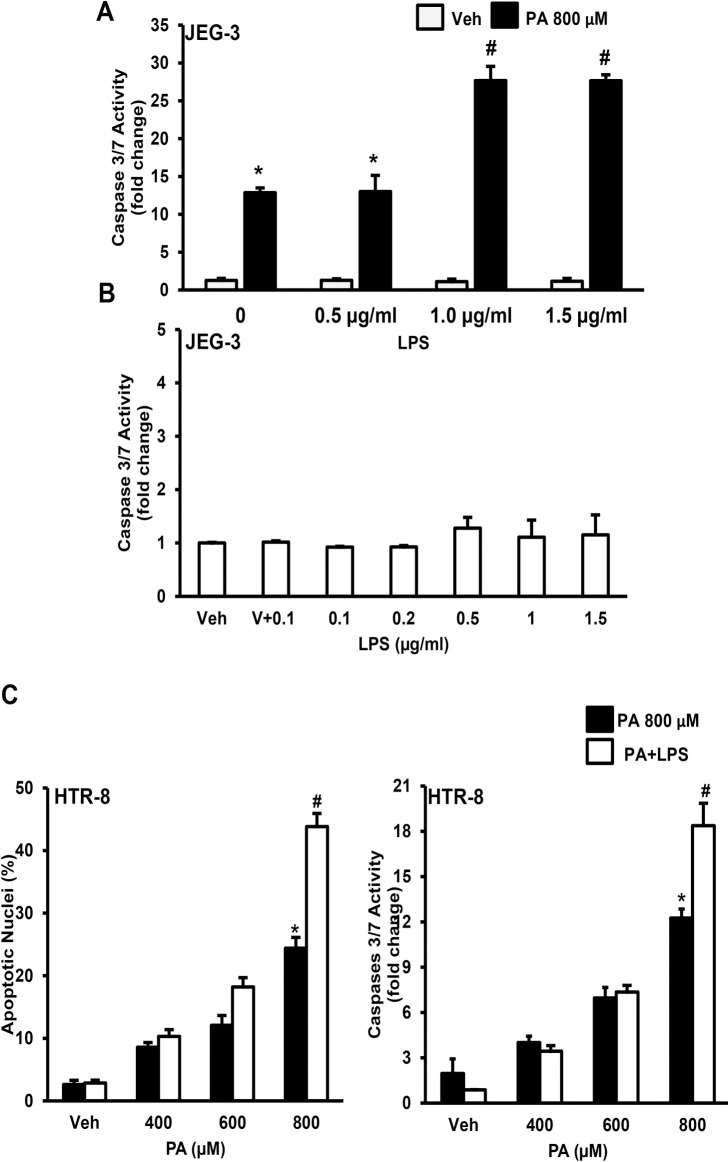
LPS aggravates FFA-induced trophoblast lipoapoptosis. JEG-3 cells were treated with 800 μM of palmitate (PA) showed increased caspase 3/7 activity. (A) Co-treatment of PA with physiological concentration of LPS (1.0–1.5 μg/ml) results in aggravation of PA-induced trophoblast lipoapoptosis; (B) JEG-3 cells treated with LPS alone (0.1–1.5 μg/ml) did not alter caspase 3/7 activity compared to vehicle (Veh) treated cells; (C) Percent apoptotic nuclei and caspase 3/7 activity were elevated in HTR-8 cells with co-treatment of PA (800 μM) and LPS (1.0 μg/mL) compared to 800 μM of palmitate alone treated cells. These data represent the mean ± sem, n = 4 and *P<0.05 compared to vehicle, #p<0.05 compared to PA alone treatment.

We treated trophoblast cells with 800 μM of PA plus LPS (0.5–1.5 μg/ml). Co-treatment of LPS (1.0–1.5 μg/ml) aggravates PA-induced trophoblast lipoapoptosis (**Fig 4A**). However, treatment of LPS alone (0.1–1.5 μg/ml) to placental trophoblast cells did not induce caspase 3/7 activation (**Fig 4B**). Further, co-treatment of LPS (1.0 μg/mL) with 800 μM of palmitate also showed enhanced caspase 3/7 activity and percent apoptotic nuclei levels compared to PA alone in HTR-8 cells (**Fig 4C**). However, co-treatment of LPS (1.0 μg/mL) with lower concentrations (400–600 μM) of palmitate for 24 h did not alter PA-induced trophoblast lipoapoptosis (**Fig 4C**). In summary, LPS exacerbates high levels of palmitate-induced placental trophoblast lipoapoptosis.

### Monounsaturated fatty acids prevents trophoblast lipoapoptosis

**Fig 5:**
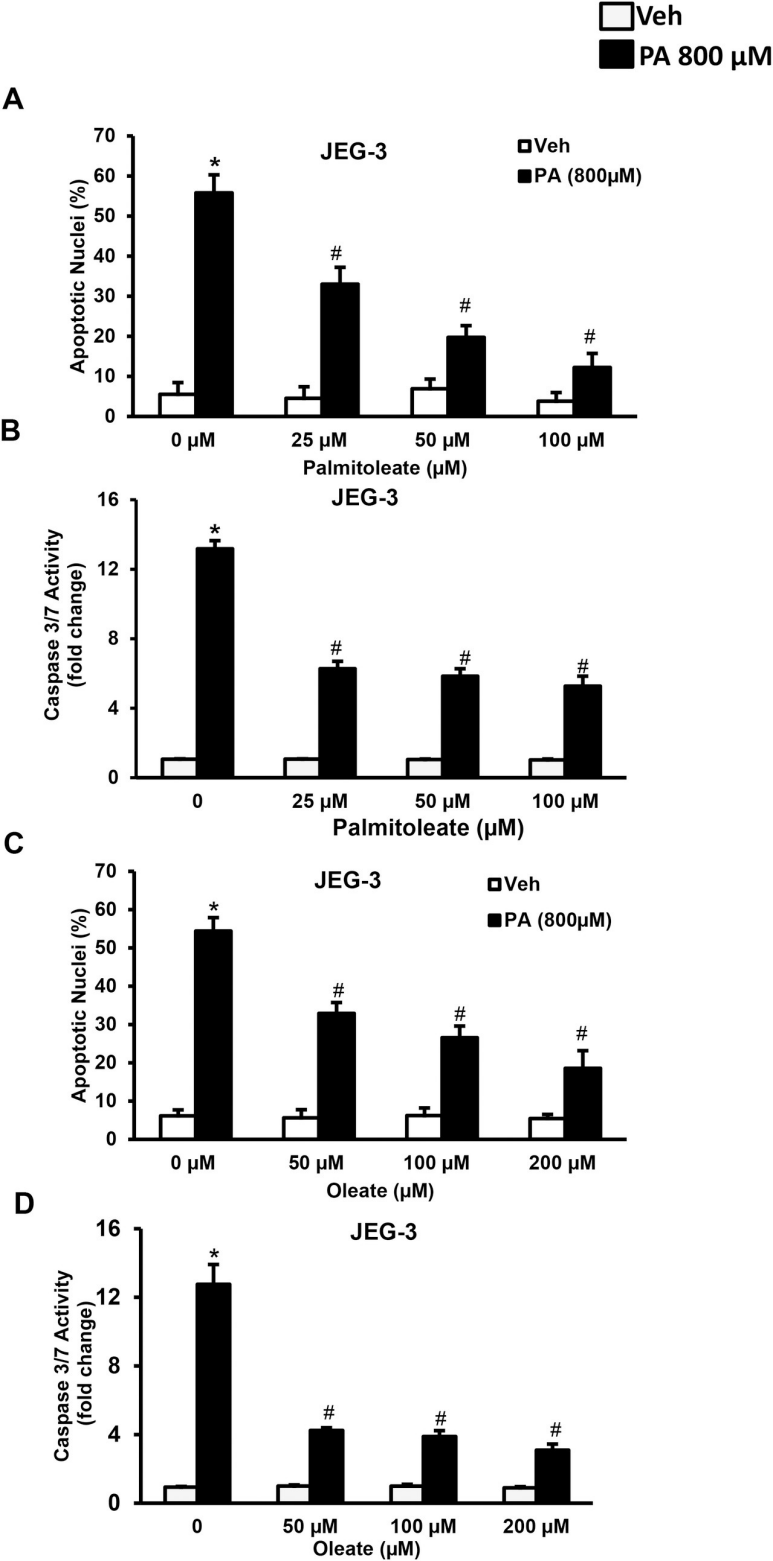
Monounsaturated fatty acids protect against palmitate (PA)-induced trophoblast lipoapoptosis. JEG-3 cells were treated with either palmitoleate or oleate alone or in combination with palmitate (PA, 800 μM) for 24 hr. (A-B) PA-induced an increase in percent apoptotic nuclei levels and caspase 3/7 activity were significantly reduced with the co-treatment of palmitoleate in JEG-3 cells. (C-D) We also observed a similar protection against PA-induced trophoblast lipoapoptosis with oleate treatment in JEG-3 cells. These data represent the mean ± sem for n = 3. * p<0.05, compared to PA (800 μM) alone treated cells, #p<0.05 compared to PA alone treatment.

Here, we treated JEG-3 cells with 800 μM of palmitate for 24 h and tested the protective role of palmitoleate or oleate. Treatment of palmitate to placental trophoblasts resulted in a significant increase in trophoblast lipoapoptosis as evidenced by an increase in percent apoptotic nuclei and caspase 3/7 activity (**Fig 5A and 5B**). Co-treatment of palmitoleate (25–100 μM) with 800 μM of palmitate for 24 h significantly protected against palmitate-induced trophoblast lipoapoptosis (**Fig 5A and 5B**). Similarly, co-treatment of oleate (50–200 μM) also significantly prevents palmitate-induced placental trophoblast lipoapoptosis (**Fig 5C and 5D**). The protection offered by palmitoleate and oleate support the therapeutic potential of monounsaturated fatty acids against placental injury and lipotoxicity during maternal obesity.

## Discussion

Increased saturated FFAs in pre-pregnancy obesity and maternal obesity could result in placental lipotoxicity; we observed that saturated FFAs induce placental trophoblast lipoapoptosis. The principal findings of this manuscript are: 1) saturated FFAs induce placental trophoblast apoptosis via caspase 3 and 7 activation, 2) LPS aggravates FFA-induced placental trophoblast lipotoxicity, *in vitro*, and 3) co-treatment of monounsaturated fatty acids like palmitoleate and oleate protects against FFA-induced placental trophoblast lipoapoptosis.

Increased placental lipotoxicity associated with decreased FA oxidation were observed in the placenta of obese mothers compared to non-obese mothers [2, 28]. Palmitate, a predominant circulating FFA exposure to placental trophoblast has been reported to induce inflammation, activation of mitogen activated protein kinase and immediate early response transcription factors like activating transcription factor 3, cJUN and serum response factor recruitment to early growth response protein 1 (EGR1) promoter [6, 17, 29]. Palmitate-induced activation of EGR1 expression and inflammation were shown to be dependent of JNK activation in placental trophoblasts. Further, obese term placentas were also shown to have increased expression of EGR1 and JNK activation [6, 17]. Palmitate-induced activation of caspases and JNK has been shown to play a critical role in hepatocyte, coronary artery endothelial cells and pancreatic-β cell lipoapoptosis [30–32]. In the present study, we show evidence for caspase activation and apoptosis with FFA exposure in placental trophoblast. To further validate the activation of caspase 3 and 7 in placental trophoblasts, we demonstrate the increased levels of caspase cleaved products suggesting the role of caspases in the mechanism of lipoapoptosis. However further investigations are required to elucidate the critical role for JNK activation in palmitate-induced trophoblast lipoapoptosis.

FFA-induced placental trophoblast lipoapoptosis was also evident in normal human immortalized first trimester-derived placental trophoblast cells (HTR-8) and term-derived placental trophoblast cells (JEG-3 and JAR cells). We observed that 400-800 μM of stearate and 600–800 μM of palmitate showed consistently increased lipoapoptosis in all three placental trophoblast cells. However, treatment of 400 μM of palmitate showed increased lipoapoptosis in JEG-3 cells, were not observed in JAR and HTR-8 cells. Increased sensitivity of palmitate-induced lipoapoptosis in JEG-3 cells should be compared with primary term-derived trophoblasts and requires further investigation. In the present study, we also observed that exposure of LPS aggravates the FFA-induced trophoblast lipoapoptosis. This data suggests that increased bacterial endotoxin, LPS and increased FFAs in maternal circulation of obese mothers can damage placental cells through multiple mechanisms of injury such as LPS-induced inflammatory changes [33] and trophoblast lipoapoptosis. Further studies are required to clearly understand the complex role of LPS-induced inflammation and TLR4 activation with FFA-induced lipotoxicity in placental trophoblasts.

Co-treatment of monounsaturated fatty acids like palmitoleate or oleate prevents FFA-induced placental trophoblast lipoapoptosis. Treatment of palmitoleate has been shown to abolish palmitate-induced endoplasmic reticulum (ER) stress response, lipotoxicity, and activation of JNK in the hepatocytes [34]. The mechanism of palmitoleate protection against hepatocyte lipoapoptosis is via abrogating the activation mitochondrial pro-apoptotic mediators like BAX, PUMA and BIM [34]. Palmitoleate is also protective against high-fat diet-induced decreased mitochondrial oxidative metabolism and activation of proinflammatory cytokines in the macrophages, respectively [35]. Further, palmitoleate and oleate supplementation results in increased expression of stearoyl CoA-desaturase 1 (SCD1), an enzyme that can convert saturated FFAs like palmitate into palmitoleate or stearate into oleate and can decrease the lipotoxicity of saturated FFAs [34, 36]. In addition, decreased expression of SCD1 enhances palmitate-induced trophoblast lipotoxicity [23] and biosynthesis of palmitoleate is decreased in the syncytiotrophoblast obtained from obese mothers compared to normal healthy mothers [22]. Recently, palmitoleate was also shown to inhibit TNFα, IL-1β and IL-6 proinflammatory cytokine responses in candida-infection stimulation analysis [37]. In the present study, we show a dramatic decrease in FFA-induced placental trophoblast lipoapoptosis with the co-treatment of palmitoleate or oleate. These data suggest that monounsaturated fatty acids like palmitoleate and oleate are anti-inflammatory and anti-apoptotic dietary nutrient molecule. However further studies are required to test the protective mechanism of monounsaturated fatty acids against saturated FFA-induced lipotoxicity.

Maternal circulating levels of triglycerides and phospho-lipoprotein lipase activity were reported to be increased in the placenta of obese mother as compared to lean mother, and lipid droplet accumulation in the placenta was also reported to be increased in maternal obesity [28]. In addition, circulating concentrations of palmitate was shown to be significantly elevated in maternal pre-pregnancy states and during gestational weeks of 24 and 34 as compared to normal weight pregnant mothers [38]. Further, increased FFAs levels were also reported in the pregnant dams of high fat high sucrose fed animals during pregnancy and this study reported endoplasmic reticulum stress in the brain to the offspring even after weaning, suggesting the lipotoxic role of FFAs to the offspring born to obese dam [39]. In the present study, we showed that these increased FFAs could cause placental trophoblast lipoapoptosis. In contrast, pregnant obese mothers and lean mothers at term were shown to have similar increased FFA levels due to maternal lipolysis [40]. However, a longitudinal study of circulating FFAs is required at various stages of gestation in lean and obese mothers.

In conclusion, we showed that saturated FFAs induce placental trophoblast lipoapoptosis. We also observed that saturated FFAs cause a concentration-dependent increase in placental trophoblast lipoapoptosis. Further, treatment of saturated FFAs to placental trophoblasts show increased biochemical markers of apoptosis like cleaved-PARP and cleaved caspase 3. Co-treatment of palmitate with lipopolysaccharide to placental trophoblasts exacerbates palmitate-induced placental trophoblast lipoapoptosis. Interestingly, co-treatment with monounsaturated fatty acids protects against palmitate-induced placental trophoblast lipoapoptosis. The protection offered by monounsaturated fatty acids support their therapeutic potential for placental lipotoxicity during maternal obesity.

## Supporting information

S1 Raw images (PDF)

## List of abbreviations

DAPI: 4’-6-diamidino-2-phenylindole dihydrochloride
FFA: free fatty acids
PAI-1: plasminogen activator inhibitor type-1
JNK: cJun N-terminal kinase
TNFα: tumor necrosis factor alpha
PUMA: p53-upregulated modulator of apoptosis
PARP: Poly (ADP-ribose) polymerase
SEM: standard error of the mean
IUGR: intrauterine growth restriction (IUGR)
LPS: lipopolysaccharide
ER: endoplasmic reticulum
IL: interleukin
