# Supplementary material for: Saturated free fatty acids induce placental trophoblast lipoapoptosis
Source: PLoS One. 2021 Apr 22;16(4):e0249907. doi: 10.1371/journal.pone.0249907 (PMC8062006; doi:10.1371/journal.pone.0249907)
Supplement: S1 Raw images — (PDF) [file pone.0249907.s001.pdf]

Saturated Free Fatty Acids induce Placental Trophoblast Lipoapoptosis

Figure 3A

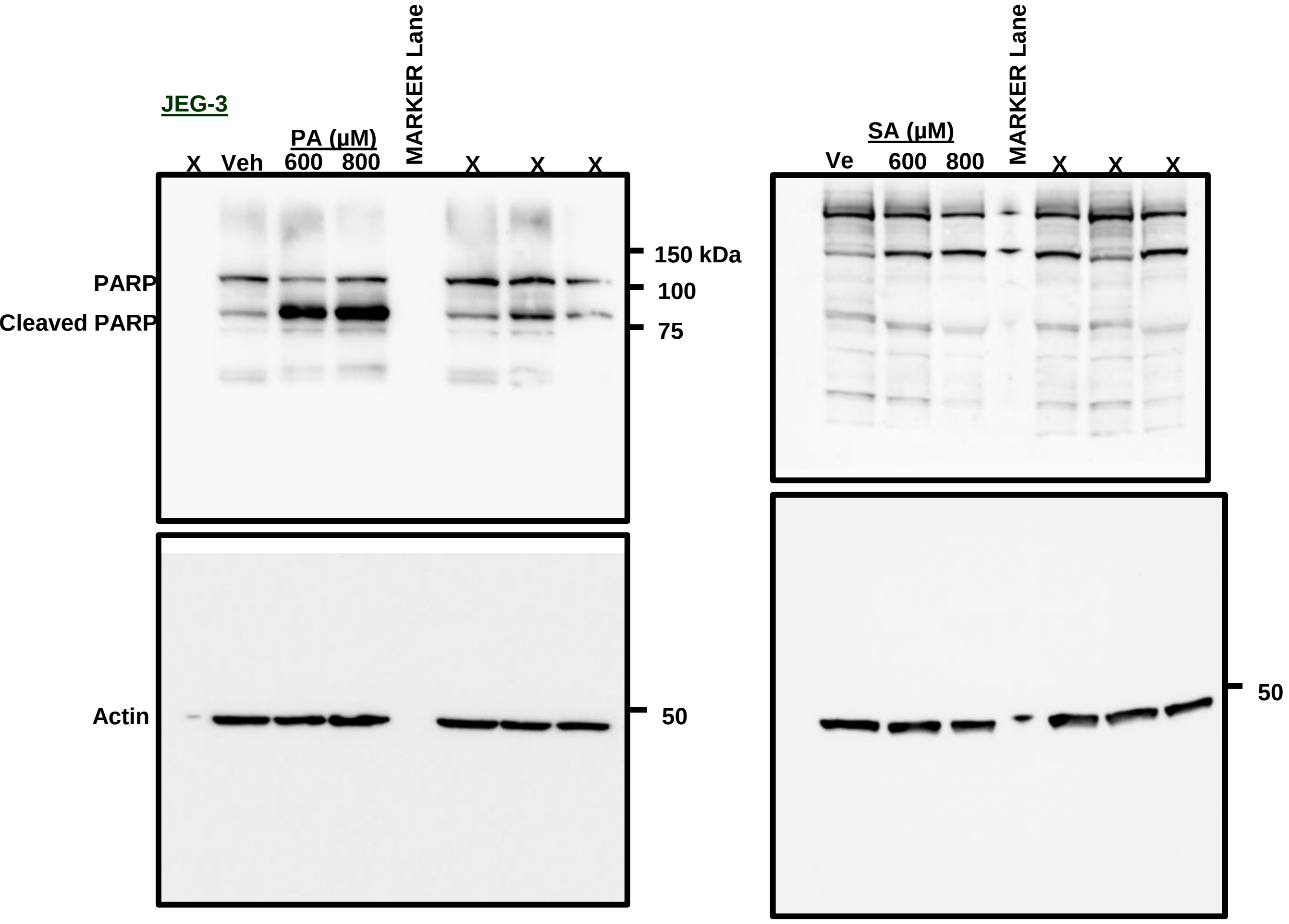

JEG-3
PA (µM)
X
Veh
600
800
MARKER Lane
X
X
X
150 kDa
100
75
PARP
Cleaved PARP
Actin
50
SA (µM)
Ve
600
800
MARKER Lane
X
X
X
50

Figure 3B

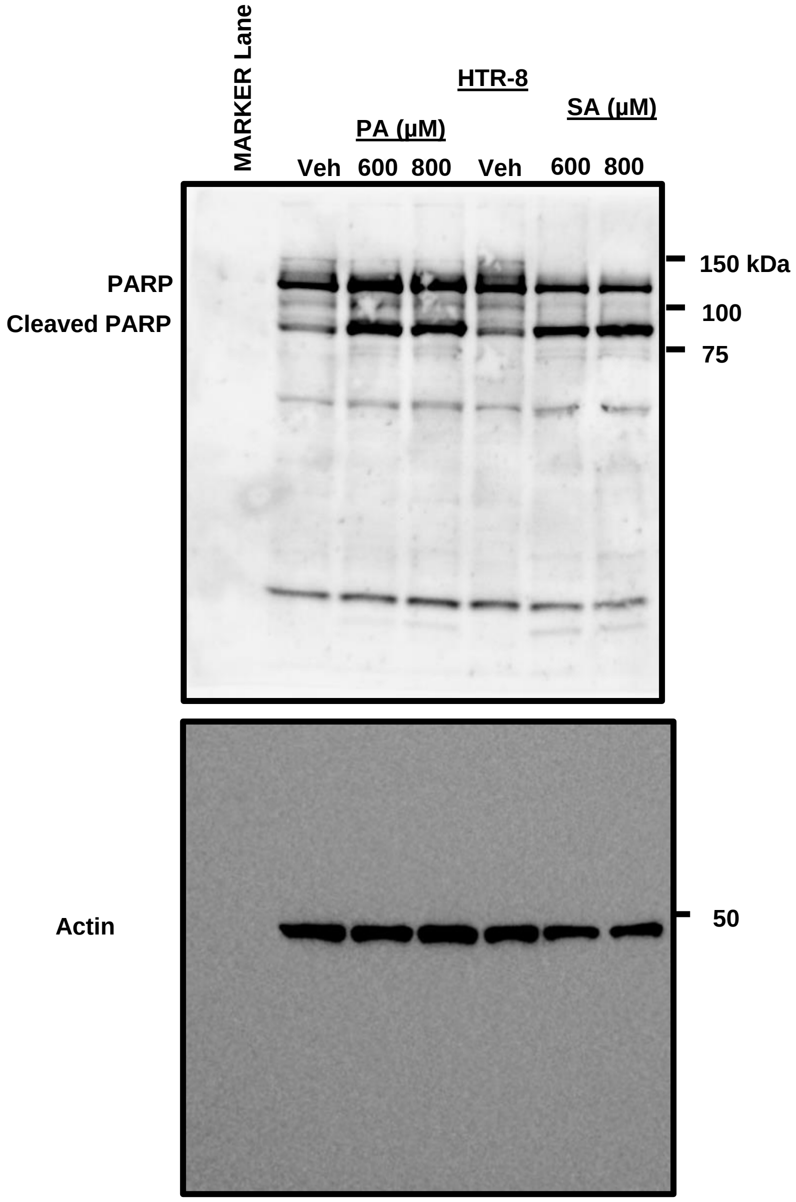

MARKER Lane
HTR-8
PA (µM)
SA (µM)
Veh
600
800
Veh
600
800
PARP
Cleaved PARP
150 kDa
100
75
Actin
50

Figure 3

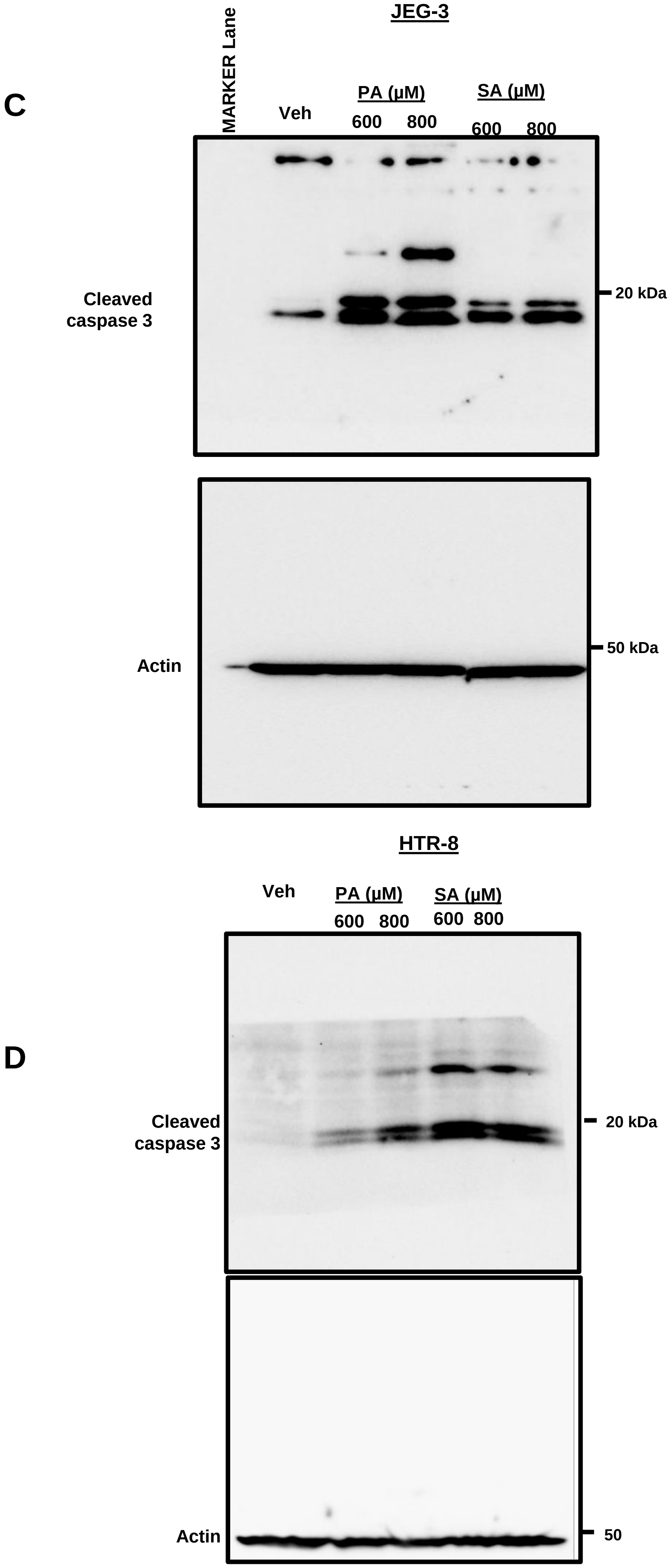
C
JEG-3
MARKER Lane
Veh
PA (μM)
600
800
SA (μM)
600
800
Cleaved
caspase 3
20 kDa
Actin
50 kDa
D
HTR-8
Veh
PA (μM)
600
800
SA (μM)
600
800
Cleaved
caspase 3
20 kDa
Actin
50
